# Bibliography

**Published:** 1914-01-15

**Authors:** 


### BIBLIOGRAPHY.

DENTAL RADIOLOGY. This is the title of a book of 200 pages published by the Swenerton Stationery Co., 121 East 27th Street, New York City. The editor is Francis L. Satterlee, Jr., A. M., D., Sc., who is lecturer on physics, etc., in the New York College of Dentistry. The book is intended as a class manual for dental students, and the text is practically a course of lectures as delivered by the author to his classes. In a very clear and practicable manner the fundamental facts in regard to the nature of the X-Ray and its application to dentistry are presented. The two uses of diagnosis and threpeusis are kept constantly in view, and so the text is made interesting as well as instructive. Naturally the diagnostic use is more definitely set forth, as it is here that the radiograph gives us light on conditions so deeply hidden in the tissues that we can by no other means know what we are dealing with. As dentists we have little difficulty in dealing with pathologic conditions when they are discovered to us. In the therapeutic realm it is probable that the different rays may yet play an important part in our treatment of peridental diseases. The author does wisely also to warn us against the abuse of this powerful agent for harm as well as usefulness. Both operator and patient, are liable to injury from overdosing or too frequent exposures. The book will be a valuable addition to our literature and ought to become popular as a college text book as well as a work of instruction for practioners who are taking up this line of work.

